# Assessment of myocardial perfusion reserve with blood oxygen level-dependent cardiovascular magnetic resonance imaging

**DOI:** 10.1186/1532-429X-11-S1-O85

**Published:** 2009-01-28

**Authors:** Jacqueline A Flewitt, Matthias Vöhringer, Jordin Green, Matthias Friedrich

**Affiliations:** 1 Stephenson CMR Centre, Calgary, AB Canada; 2 Robert-Bosch-Krankenhaus, Stuttgart, Germany; 3 Seimens Healthcare Canada, Calgary, AB Canada

**Keywords:** Myocardial Perfusion, Myocardial Blood Flow, Coronary Sinus, Adenosine Infusion, Cardiovascular Magnetic Resonance Image

## Background

New Blood Oxygen Level-Dependent Cardiovascular Magnetic Resonance Imaging (BOLD-CMR) sequences show a high sensitivity and consistent image quality that allows for assessing tissue oxygenation. We hypothesized that BOLD-CMR can quantitatively assess myocardial blood flow changes using myocardial oxygenation as a biomarker.

## Objective

To test whether a BOLD-CMR sequence accurately estimates myocardial perfusion changes.

## Methods

Six anesthetized mongrel dogs were instrumented with a coronary infusion catheter in the circumflex coronary artery (LCX), an MR-compatible epivascular flow probe around the LCX and a catheter in the coronary sinus. Using a clinical 1.5 T MRI system (MAGNETOM Avanto, Siemens Healthcare, Germany), SSFP BOLD-CMR was performed during graded intracoronary infusion of adenosine in the LCX. Typical scan parameters were: Field-of view (FOV) 190 × 280 mm; matrix size 106 × 192; slice thickness 10 mm; T_R_/T_E_ 5.8/2.9 ms; flip angle 90°; typical breath-hold duration 14 s. Images were analyzed using clinically validated software (cmr^42^, Circle Cardiovascular Imaging Inc., Calgary, Canada) and the BOLD signal intensity (SI) for each was calculated. Correlations of coronary flow, oxygen saturation in the coronary sinus and myocardial BOLD-CMR signal intensity (BOLD-SI) changes were calculated by regression analysis. The same CMR imaging protocol was used in 11 healthy volunteers (6 male, 5 female) before, during and after intravenous adenosine infusion (140 micro-g/kg). Myocardial perfusion reserve in the human volunteers was calculated from flow measurement in the coronary sinus using velocity-encoded CMR.

## Results

**Figure 1:**
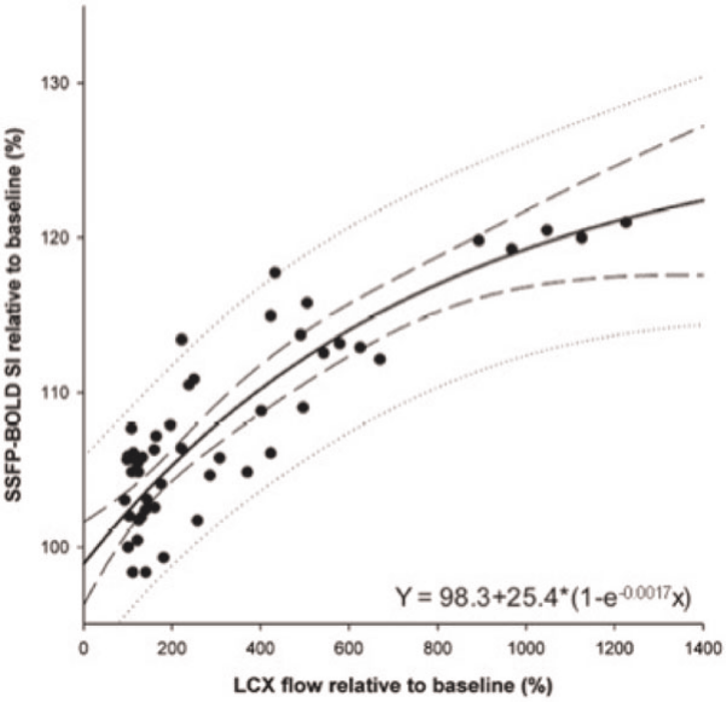
**Blood flow changes and BOLD-SI in canine model under adenosis infusion**.

In dogs, adenosine-induced blood flow changes in the LCX agreed very well with changes in coronary venous saturation (logarithmic scale, r^2^ = 0.94, p < 0.001). Furthermore, coronary venous saturation showed a strong yet linear correlation with BOLD-SI changes (r^2^ = 0.80, p < 0.001). Consequently, as shown in Figure 1, blood flow changes correlated very well with the BOLD-SI (r^2^ = 0.84, p < 0.001). The exponential correlation is described by the equation (y) = 98.3+25.4*(1-e^-0.0017x^) (x = flow, y = BOLD-SI). In the volunteers, adenosine infusion resulted in a significant myocardial perfusion increase (416 ± 69% of baseline, p < 0.001). BOLD SI increased significantly by 20.1 ± 9.5% (p < 0.001 as compared to baseline). The reproducibility of the BOLD-SI in the two baseline measurements before and after adenosine infusion was excellent (mean difference 0.1 ± 2.6%, p = 0.97).

## Conclusion

State-of-the-art BOLD-sensitive MRI sequences detect changes of myocardial perfusion in an experimental animal model and in humans in vivo. This technique may allow for an accurate, non-invasive assessment of myocardial perfusion reserve in humans.

